# Hepatitis C Micro-Elimination beyond Prison Walls: Navigator-Assisted Test-and-Treat Strategy for Subjects Serving Non-Custodial Sentences

**DOI:** 10.3390/diagnostics11050877

**Published:** 2021-05-14

**Authors:** Joaquin Cabezas, Susana Llerena, Miguel Mateo, Rocío Álvarez, Carmen Cobo, Victoria González, Elisa Martró, Antonio Cuadrado, Javier Crespo

**Affiliations:** 1 Gastroenterology and Hepatology Department, University Hospital Marques de Valdecilla, Research Institute Valdecilla (IDIVAL in Spanish), 39008 Santander, Spain; joaquin.cabezas@scsalud.es (J.C.); susanallerena@hotmail.com (S.L.); aculavin@hotmail.com (A.C.); 2 Medical Department, “José Hierro” Social Integration Unit Health Centre, 39011 Santander, Spain; miguel.mateo.soler@gmail.com (M.M.); rocio.alvarez@dgip.mir.es (R.Á.); 3 Medical Service, “El Dueso” Penitentiary Centre, 39740 Santoña, Spain; carmencobopelayo@hotmail.com; 4 Centre of Epidemiological Studies of HIV/AIDS and STI of Catalonia (CEEISCAT)—Catalan Institute of Oncology (ICO)—Public Health Agency of Catalonia (ASPCAT), 08005 Badalona, Spain; vgsoler@iconcologia.net; 5 Microbiology Department, Laboratori Clínic Metropolitana Nord, Hospital Universitari Germans Trias i Pujol, Germans Trias i Pujol Research Institute (IGTP), 08916 Badalona, Spain; emartro@igtp.cat; 6 Centro de Investigación Biomédica en Red en Epidemiología y Salud Pública (CIBERESP), Instituto de Salud Carlos III, 28029 Madrid, Spain

**Keywords:** hepatitis C, underserved, navigator, point-of-care test, community sentences, correctional setting

## Abstract

Background and Aims: The Spanish prison population includes two groups: people in prison and those who are serving non-custodial sentences. The latter has not yet been studied. This study aims to describe this population and the results of a test-and-treat strategy for hepatitis C including a holistic health assessment. Method: This prospective study included all subjects serving non-custodial sentences at the Center for Social Integration. It was assisted by the medical team, a navigator, and a systematic screening of HCV (Hepatitis C Virus) performed by point-of-care tests. All cases with active infection are evaluated using telemedicine by a specialist to prescribe antiviral treatment. The navigator facilitates continuity for medical and social assistance. Results: The screening rate reached 92.8% (548/590). HCV seroprevalence and viraemia prevalence were 8% (44) and 2.9% (16), respectively. Regarding comorbidities: problems related to drug dependence were detected in 264 (48.2%), suspected serious mental disorder in 44 (8.3%), and previous stay in prison in 122 cases (22.2%). The navigator monitored 59 (15.2%) patients regarding HCV treatment or comorbidities. All patients (10/10) completing 12 weeks follow-up achieved sustained virological response. Conclusions: The population serving non-custodial sentences is a challenging group with a high prevalence of HCV infection. Micro-elimination programs using point of care diagnostic tests, telemedicine, and a navigator are necessary in this underserved vulnerable population.

## 1. Introduction

Being the major cause of liver-related morbidity and mortality, the Hepatitis C virus (HCV) infection shows a considerable humanitarian and medical services concern internationally. Notwithstanding, a feasible chance of cure exists, with specific targets for the definitive eradication of HCV infection as health menace for the population by 2030 [1,2,3]. The elimination targets are attainable in developed countries due to the extensive accessibility of direct-acting antiviral agents (DAAs) [4]. As a result of this global agreement, different governments in Western countries have implemented mechanisms of action at different levels of infection that have resulted in the elaboration of specific action plans against hepatitis C. In the particular case of Spain, the recent National Strategic Plan against Hepatitis C (PEAHC) has established different lines of action against hepatitis C providing economic resources at budget level for its implementation [5]. As a result of these actions, approximately 100,000 patients have been cured with direct action antivirals (DAA) since 2015 in Spain. Furthermore, stricter and more accurate screening methods such as reflex RNA testing have been established [6].

Recently, a prevalence of active infection (detectable viremia) of 0.31% has been detected, which is significantly lower than the 400,000 viremic patients (0.85%) initially estimated [7]. A recent population-based study has reported a viremic prevalence in Spain of 0.49% in a population 25–70 years old [8]. In summary, there are between 100,000 and 150,000 viremic patients in Spain that are susceptible of being treated and more importantly, up to 30–50% of them are unaware of their infection and/or are even out of the health system (i.e., people who inject drugs (PWID), people in prison, men who have sex with men (MSM), people suffering from mental disorders, etc.,).

However, there are still some problems to deal with before the objective of HCV elimination in the frontier of the 2030 can be achieved. On the one hand, there are some countries that still face budgetary barriers to carry out large-scale population-based programs of screening and treatment. On the other hand, developed countries (like Spain, the Netherlands or Germany) with high DAA treatment rates fight to prevent “diagnostic burn-out” (a phenomenon highlighted by Hill et al. [9], in 2017) by focusing screening on the most at-risk populations. These researchers used a Markov model in which given the low annual rates of current diagnostic concluded that in 2022 Spain could reach a phenomenon of “diagnosis exhaustion” of HCV.

As a result of these drawbacks and threats, a group of experts headed by Prof. Lazarus have recently promoted micro-elimination policies as a mean of dealing with HCV elimination in different settings and regions [10,11]. This concept is based on the idea that the most pragmatic approach would be to break down national HCV-elimination goals to achieve the WHO targets into smaller goals by acting on specific sub-populations (e.g., people living with HIV, people in prison, hemophiliacs), settings (hospitals, drug dependence treatment centers), generational cohorts (baby-boomers), or geographic areas. In line with this approach, other authors and even the aforementioned PEAHC have given priority to prisons and other closed settings as a target population that must be prioritized [12].

About 30% of HCV infections are from people in prison [13]. The prison setting tends to concentrate HCV infection, showing a prevalence up to ten times the community (17–64%) [14]. Mean prevalence in Western European and US prisons is around 15% [14], and recently in a Spanish study, including 18 prisons, showed a prevalence of 22.7% [15].

One of the pioneer initiatives of micro-elimination has recently been carried out by our group in the El Dueso prison (Cantabria), the Jailfree-C project [16]. This program consists of performing a sustained strategy of HCV infection “test-and-treat” of all inmates admitted. Interestingly, this successful micro-elimination program has focused on a well-known population reservoir that concentrates mental disorders, drug dependencies, and infectious diseases that could spread in the community.

Another potential reservoir where infections and other diseases can be concentrated, and is subjected to be part of a micro-elimination program is the one composed by people who has been condemned to penalties and alternative measures that do not involve deprivation of liberty, named non-custodial sentences or community sentences. These are people who have suffered a sentence that has been commuted, at least temporarily, to community work or other community services, preventing these people from being admitted in prison. This population has acquired great importance in different European countries like in Spain. There are approximately 140,000 persons serving these types of sentences (slightly more than twice the prison population). One of the main features of this population is that their members transit very easily between the community and the prison especially when they are not adherent to the terms of the sentence. Therefore, this is a vulnerable, poorly understood population that has traditionally escaped from the scope of Health policies and research. However, some of the characteristics of this population in Spain have been described (i.e., predominantly male, most of them between 26 and 35 years-old, 81% Spanish, up to 70% have studies below the compulsory education, and only 30% are active workers; unpublished data, personal communication). It is very likely that a high percentage of this population concentrates disorders like viral infections, psychiatric illnesses, and drug dependences as it also occurs in people in prison. These people are attended in Social Integration Centers (SIC), and these sentences depend on Department of Sentence Management and Alternative Measures Services that are integrated in the structure of the General Secretariat of Penitentiary Institutions (Ministry of Interior of the Spanish Government). This type of penalties keeps the offender within their community environment, subjected to certain restrictions, conditions, or obligations, depending on the case. The José Hierro SIC in Santander-Cantabria is one of the 32 SIC distributed in the Spanish geography. The center has medical staff, psychologists, social services, specialists in drug dependence, etc.

We have designed a project, named The HONEST (A novel Hepatitis c micrO-elimination program in Non imprisonEd SenTenced with alternative measures) project, which is focused on this especially vulnerable population, with mental disabilities, drug dependences, or social exclusion problems.

The HONEST project is aimed to link people attended at SIC José Hierro to the Chronicity and Mental Plans of the Cantabria’s Government taking advantage of an HCV micro-elimination program. This project is aimed to uncover this volatile population and to engage these users to the health system taking advantage of these tools. Finally, this model of care includes point-of-care testing (POCT) by a single capillary sample at the SIC to determine prevalence of viral infections, and then a global and quick health evaluation supported by a navigator and a telemedicine tool.

## 2. Materials and Methods

### 2.1. Study Design and Population

This is a prospective study, including all subjects serving non-custodial sentences (Clinicaltrials.gov Identifier: NCT03932396) attended at the Social Integration Center (SIC) “José Hierro” (Santander-Spain) from June 2019 to June 2021. All subjects attending the SIC who were between 18 and 79 years and who gave signed informed consent could be enrolled as the only eligibility criteria.

### 2.2. Phases and Professional Network

**Figure 1:**
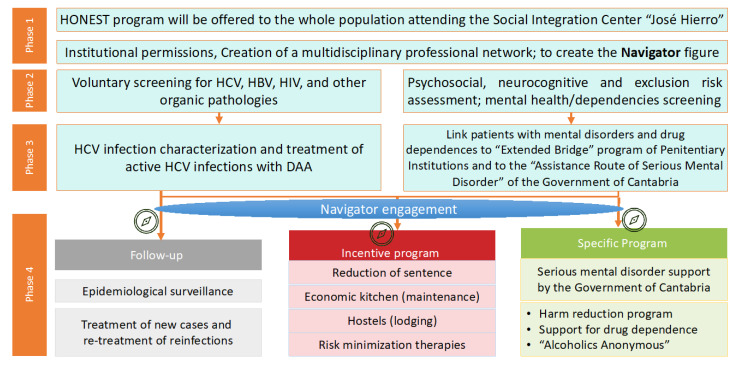
Phases of the study. Detailed design of the project.

Following our previous experience in the prison setting [16], we carried out the study with a multidisciplinary team at SIC end and Valdecilla University Hospital. It included four phases depicted in Figure 1. In this setting, we created a new figure we named “Navigator.” There are some similar experiences [17] recently published. We generated this figure, the Navigator to serve as a support at the correctional setting to set a continuum of health attention to subjects and engage them with global health evaluation, hepatitis C screening, and linkage to care. In our region and widely in Spain [6], HCV diagnosis is performed with a single blood sample, which is called reflex test: this means that once the HCV antibody is detected positive, automatically a RNA detection is performed in the same blood sample. In this setting, we incorporated point of care tests to detect active infection in a single visit from capillary blood. First, antibodies to HCV were detected with Oraquick^®^ HCV Rapid Antibody test (OraSure Technologies, Bethlehem, PA, USA) in capillary blood and, if positive, the Xpert^®^ HCV VL Fingerstick assay was performed on a Gene Xpert^®^ System (Cepheid, Sunnyvale, CA, USA) to quantify HCV RNA.

In this first approach, variables including anthropometrics, demographics, risk behaviors, medical history, particularly if related to HCV infection and psychiatric conditions, were recorded. Co-infection with HIV was assessed in the laboratory by the Murex HIV antigen/antibody combination assay (DiaSorin, Crescentino, Saluggia VC, Italy) from dried blood spots as previously described [18].

In a second visit at the hospital end, a complete liver disease evaluation was performed (including complete blood test to rule other liver diseases, Fibro-Scan^®^-EchosensTM NorthAmerica; 1050 Winter Street, Waltham, MA, USA, and an ultrasonography if cirrhosis was stated). Population included in the study was followed to detect new infection or re-infection every 6 months when serving the sentence or were encouraged to keep the attention in other facilities (addiction centers, primary care ambulatories).

### 2.3. Recruitment Strategy

Subjects sentenced to non-custodial sentences are required to check-in in the SIC when they receive a letter that states, they start to serve their sentence. This letter includes an invitation to accept a health evaluation. This global health care assessment is performed by the medical staff and/or navigator in the SIC and includes a HCV screening test as explained above. Once the person is informed and signs the consent, the health evaluation is done.

To improve the enrollment in this model of care an incentive program was included. Navigator and medical staff in the SIC provided lunch and travel tickets to the participants. Other key point in this incentive program is the possibility to serve the sentence if the subject is compliant with the health care appointments and attends their visits and treatments (Article 5 of Royal Decree 840/17 June 2011); i.e., the time spent attending the health programs is reduced from the time of the initial sentence.

### 2.4. Intervention

**Figure 2:**
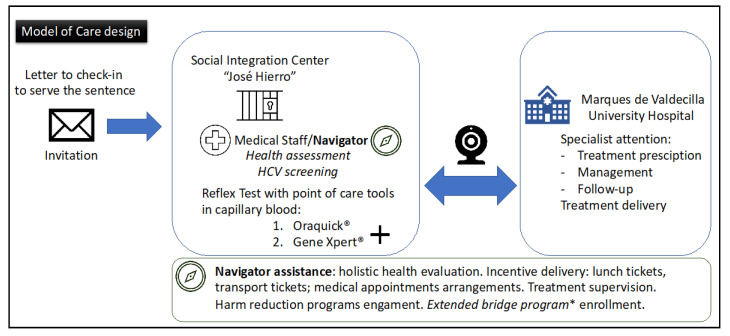
Model of care design. Social Integration Center “José Hierro” (SIC)—it is the location where these subjects check in to serve these sentences. Extended Bridge program*: health program supported by the government included in a global plan to attend chronic diseases, this one is focused on severe mental illness to serve sentences with alternative measures.

We designed a model of care (Figure 2) assisted by the medical team and the center’s “Navigator”, based on systematic screening of HCV by detection of antibodies with Oraquick^®^, for those individuals that test positive, viral load is determined by Gene Xpert^®^ in capillary blood. Gene Xpert^®^ System was installed at the SIC. Cepheid^®^ delegates taught medical staff how to carry out the tests, to get the blood sample, and how to set up and run the machine. Nurse and physician at the SIC performed all the tests.

All cases with detectable viral load are evaluated by the hepatology/infectious disease staff by telemedicine and then antiviral treatment is prescribed. Thus, active HCV infection detection, disease evaluation, and treatment are done in the same day. Treatment was prescribed at the discretion of the physician following current guidelines [4]. Telemedicine was carried out as we previously did in the JailFree-C project [16,19]. SARA network support Reunete is the application to carry out teleconsultations. Teleconsultation was carried out with the patient and medical staff of SIC in one end and the specialist in the hospital end.

The Navigator facilitates continuity for medical care and social assistance of these individuals (accompaniment to the hospital, adherence to treatment etc.,).

### 2.5. Ethics

The direction of the study was authorized by all Institutional Review Boards (IRB) and by the Ethics Committee of Cantabria (Identification number: 2019,025; act 03/2019 of 15 February 2019) in accordance with the Declaration of Helsinki and International Conference on Harmonization established instructions. The trial was monitored by an accountable assigned individual from the IRB with the aim of guaranteeing self-reliance, self-determination, and lawfulness in this setting. A programming of subsequent regular visits to the investigators and even the social integration center occurred. All through the examination, members were allowed to pose inquiries to the specialists and to decline their participation with no repercussions in the sentence time frame. The signature of a written informed consent was provided to every patient included before admission.

### 2.6. Statistical Analysis

A descriptive analysis was accomplished. Categorical variables were described with percentages, and continuous variables were depicted with mean and standard deviation or median and range/interquartile range (IQR) as appropriate. The statistical analysis was performed with SPSS Statistics for Windows, Version 25.0 (IBM Corp, Chicago, Armonk, NY, USA). All *p*-values were two-tailed. Statistical significance was defined as *p* < 0.05.

## 3. Results

### 3.1. Characteristics of the Study Population

**Figure 3:**
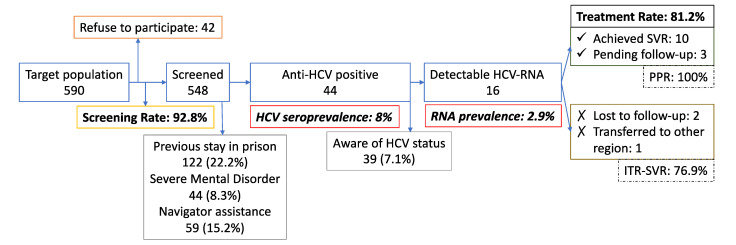
Flow chart of the study. SVR: sustained virological response (negative HCV RNA 12 weeks after the end of treatment). PPR: per-protocol response. ITR: intention to treat response.

All patients attending SIC were invited to this program, 590 subjects were sentenced to non-custodial penalties in the study period. We screened 548 subjects, resulting in a 98.2% screening rate (See Figure 3). Most of them were male (86.5%) and below 60 years-old (94.3%) with a median age of 38 years-old (range 18–76), few of them with high educational level (2.9%) and frequently unemployed (48.9%). Many of the patients recognized drug consumption; 48% (264/548) had drug dependence, including both legal and illicit substances, (tobacco/alcohol for more than a half, and cannabis or cocaine for one out of three). The use of these drugs was significantly more frequent in the HCV actively infected cohort. Furthermore, a previous stay in prison and intravenous drug use were significant risk factors for HCV infection in this group. We also found 8.3% of subjects suffering from suspected severe mental disorders in their initial evaluation. Table 1 shows the demographic, clinical, and infection features of both the whole cohort and those with a viremic infection. HCV antibodies were present in 8% (44/548) (CI 95%: 6.03–10.6). Overall, 2.9% (16) (CI 95%: 1.8–4.7) had detectable HCV-RNA (2 of them were HIV positive). Only 7% of the whole cohort knew their HCV serological status. Overall, 36.3% of seropositive participants were viremic. Half of the patients had HCV genotype results available in their clinical records, a third of them were infected with genotype 1. They presented mild to moderate fibrosis stage (F0–2: 61.5%), although 1 out of 3 presented with advanced fibrosis, close to 25% were cirrhotic. None of the latter showed any liver decompensation at the diagnosis or during the follow-up, as well as no hepatocellular carcinoma was detected.

A brief summary of the offences type and sentences that this population is serving is included in Table S1. Most of these offenders are serving sentences with works for the community benefit due to gender violence and restraining order infringement.

### 3.2. Efficacy

All patients with detectable RNA were prescribed antiviral treatment, except for one—an HIV co-infected patient who did not remember his anti-retroviral treatment, hence drug–drug interaction evaluation could not be checked during teleconsultation. Finally, this patient was transferred to another region and did not start treatment within this program. Two patients did not show up to take the treatment after prescription and were lost to follow-up. This resulted in a treatment rate of 81.2%. At the time of writing, 10 patients had completed follow-up and 3 were awaiting the SVR visit. Thus, we found 100% SVR (CI 95%: 72.2–100) for those completing the protocol, however intention to treat response was 76.9% (10/13) (CI 95%: 76.9–91.8). Figure 3 depicts these results.

### 3.3. Navigator

This figure, a psychologist, was integrated with medical staff at SIC and carried out global health assessment. Following navigator evaluation, 69 patients (12.6%) were referred to Mental Health Unit (Extended Bridge Program), 37 (6.7%) to drug dependence treatment centers, and 18 (3.3%) received social assistance (application for economic benefit, work or recognition of disability). Besides, the navigator assisted 59 (15.2%) patients during the program specifically related to HCV management: attention to medical, tests and treatment delivery appointments, and antiviral adherence.

## 4. Discussion

This is the first study that draws attention to a novel underserved population within the penitentiary setting, serving non-custodial sentences, who show a high prevalence of HCV infection. We found 2.9% of these subjects were viremic, which represents a prevalence of active infection ten times higher than that in the community [7].

Similarly, to those in prison [16], they are a complex population suffering from drug dependence and their consequences showing a high proportion of mental disorders, as we found 48% of drug dependence and 8% of severe psychiatric conditions.

Fortunately, implementing in this setting a multidisciplinary team together with a navigator and a telemedicine tool, and following a test-and-treat strategy, we were able to screen more than 90% of the attendees to SIC, treat more than 80% of patients with an active infection, and finally reaching HCV cure in all patients completing treatment.

Little is known about the people sentenced to non-custodial sentences [20,21], and these studies are not focused on infectious disease assessment, despite a clear presence of risk factors. Alternatives to incarceration [22] are better measures to favor integration and rehabilitation and they can prevent from prison admissions, thus reducing stigma. This is also relevant when these types of sentences are coupled with a holistic health evaluation, linking people with their needs, such as mental health, dependence care centers, employment assessment, and so on.

To provide a global health attention our administration is running two plans: Chronicity Attention Program and the Mental Health Plan of the Cantabria’s Autonomous Government. These plans are two cornerstones of the health policy of the Government of Cantabria and prioritize the care of the person with severe and chronic mental illness and disorders related to the abuse of alcohol and other substances. One of the actions of these programs is called the Serious Mental Disorder Care Path, which aims to identify and respond to all patients with severe and chronic mental illness in an efficient and quality manner. Interestingly, the program “Extended Bridge” from Penitentiary Institutions is also devoted to deal with people with severe mental disorder who can meet an alternative criminal measure through participation in programs and aims to detect early mental disorders in people prosecuted, improving their health and attachment to socio-health devices; at the same time, it helps them to avoid the consequences derived from non-compliance, among which is the entry into prison.

HCV elimination efforts may be enhanced by micro-elimination policies focused on specific populations and their needs [11]. These programs should be based on decentralization [23] of diagnosis and treatment. In this sense, in Spain, this is particularly important in infection detection. After the wide access to reflex testing [6] for a routine diagnosis within the hospital or primary care setting, we are now working toward the dissemination of point-of-care tools in order to make them available to underserved populations. Saludes et al. nicely carried out HCV RNA detection with dried blood spots and GeneXpert in the harm reduction setting in Catalonia [24,25]. While dried blood testing allows for multiple testing and the detection of co-infections, these results are deferred to a second visit. Our model, including Oraquick and GeneXpert carried out with capillary blood provides results in the same day. This way of performing a reflex test for HCV has already been studied in the prison setting [26]. Time while test is running was used to engage patients with a lunch ticket, and global health evaluation to rule out severe mental disorder or drug dependence problems. In case of any of these issues, the navigator referred the patient to whichever program they needed.

In the HONEST project, we were able to carry out a complete diagnosis, specialist assessment, and treatment prescription within the same visit. However, either the patient or the navigator or both had to go to the hospital pharmacy to get the antiviral treatment. Two patients did not start treatment and were lost to follow-up. Decentralizing diagnosis is possible, why not to decentralize treatment delivery? Dillon’s group carried out a trial (REACH HCV) to investigate a model of care for clients receiving opioid substitution therapy [27], they can be tested in pharmacies at the time they get methadone and start HCV treatment if they test positive. Moving the setting to harm reduction centers is the model of Lens et al. [28]. They have designed a pretty similar way of work, complete HCV infection assessment from antibody and RNA test to disease evaluation with a transient elastography and finally treatment delivery. They got a screening rate of 58%, 65% of people with an active infection started treatment and 70% achieved SVR. Finally, Valencia et al. [29] propose a mobile unit to reach underserve population to manage HCV infection. These patient-centered models of care aim to achieve HCV elimination in this setting with high-risk practices.

Recently released recommendations [30,31] and guidelines regarding Hepatitis C management [32,33] state for its simplification to improve the care cascade continuum from the access to diagnosis through a quick result and an immediate and easy treatment start.

Unluckily, this is a single center experience with no randomization and no arm to compare. Furthermore, this is the first time this population is studied, thus there is no historical attention records to compare with the previous model of care. In the past, as it happened in the drug dependence treatment centers, these people had to be referred to a primary care ambulatory to seek for HCV testing. Then, they had to wait for the result and a new referral to the hospital for specialist attention. Moreover, self-reported information on bio-behavioral data, previous testing, diagnosis, and treatment may not be completely reliable due to limited recall, stigmatization, or poor comprehension. However, we believe that this bias was minimized by the fact that the interviews were performed by trained staff within an environment of trust.

Moreover, hepatitis C treatment delivery must be done at the hospital in the end, which is still a hard barrier to overcome. Patients can be screened and evaluated by the specialist just in a few hours, however they have to move to get the treatment. This was one of the main shortcomings of this study, that could have led to two of our lost-to-follow-up patients.

In the penitentiary setting, health care depends on both administrations: Ministry of Interior Affairs and Ministry of Health (except for Catalonia and the Basque Country). This issue can sometimes make it difficult to get agreements and funding. This project is still running, and we hope to show its usefulness to continue with the program and export it to other settings.

This model of care shows great results in our region. At least, around Spain there are several similar centers with almost comparable resources than can implement this model of care. Pretty similar models are already working in addictions center and mobile clinics (Denmark) [34,35], where peers [36] help people to join these centers to take screening tests and to enroll harm reduction program and treatment facilities in Canada [37] and Mexico [38].

## 5. Conclusions

The population serving non-custodial sentences is a challenging group, difficult to approach with high-risk practices and severe mental disorders and with a high prevalence of HCV infection. Micro-elimination programs like this, using rapid diagnostic tests, telemedicine, and a “Navigator” figure are necessary in these underserved and vulnerable populations.

## Figures and Tables

**Table 1:** Main features of the study population.

*n* (%)		Screened Population (*n* = 548)	HCV RNA Positive Cases (*n* = 16)
Male		455 (86.5)	16 (100)
Age (years)	18–39	287 (54.6)	0
	40–59	209 (39.7)	16 (100)
	>60	30 (5.7)	0
Educational level	No studies	25 (6.6)	1 (6.2)
	Primary	239 (62.9)	12 (75)
	Secondary	101 (26.6)	3 (18.8)
	Superiors	15 (3.9)	0
Current job	Employed	106 (27.7)	5 (31.2)
	Unemployed	239 (62.4)	10 (62.5)
	Retired	23 (6)	0
	Inability	15 (3.9)	4 (25)
Drug consumption	Tobacco	321 (61)	16 (100)
	Alcohol	361 (68.3)	10 (62.5)
	Cannabis *	176 (33.5)	9 (69.3)
	Cocaine *	146 (27.8)	4 (30.8)
HCV risk factors	Tattoos	234 (42.7)	7 (43.8)
	Prostitution	6 (1.1)	0
	Previous stay in Prison *	122 (22.3)	10 (62.5)
	Transfusion	21 (3.8)	0
	MSM	13 (2.4)	1 (6.3)
	PWID *	11 (2)	4 (25)
Drug-use problems		264 (48.2)	10 (62.5)
Suspected severe mental disorder		44 (8.3)	3 (18.7)
HCV genotype			
ND			8 (50)
1			3 (18.8)
1a			2 (12.5)
2			1 (6.2)
3			1 (6.2)
4			1 (6.2)
Fibrosis assessment			
F0–1			7 (43.5)
F2			4 (25)
F3			2 (12.5)
F4			3 (18.8)
Co-infections			
HBV			0
HIV			2 (12.5)
Previous HCV treatment			3 (18.7)
Analytical parameters (mean)			
Total bilirubin (mg/dL)			0.5
Albumin (g/dL)			4.5
Platelets (×10^3^/µL)			180

MSM: men who have sex with men. PWID: people who inject drugs. ND: non-determined; * *p* < 0.05.

## Data Availability

Data cannot be publicly available due to confidenciality.

## Supplementary Materials

The following are available online at https://www.mdpi.com/article/10.3390/diagnostics11050877/s1. Table S1. Type of offence and sentences features of the whole population.
