# Supplementary material for: Hepatitis C Micro-Elimination beyond Prison Walls: Navigator-Assisted Test-and-Treat Strategy for Subjects Serving Non-Custodial Sentences
Source: Diagnostics (Basel). 2021 May 14;11(5):877. doi: 10.3390/diagnostics11050877 (PMC8155928; doi:10.3390/diagnostics11050877)
Supplement: Supplementary file 1 [file diagnostics-11-00877-s001.zip › diagnostics-1146937-supplementary.pdf]

**Table S1.** Type of offence and sentences features of the whole population.

| Type of Offence | Proportion of the cohort $n$ = 528 |
|---|---|
| Gender violence | 132 (25%) |
| Restraining order | 133 (24.6%) |
| Road safety offences | 112 (21.2%) |
| Sentences features | |
| Works for the community benefit | 431 (81.6%) |

There were no significant differences between those HCV positive and negative in terms of type of sentence. Mean length of the sentences was 80 h.
